# The Impact of Cultural Immersion Experience on Identity Transformation Process

**DOI:** 10.3390/ijerph18052680

**Published:** 2021-03-07

**Authors:** Gloria Onosu

**Affiliations:** Krannert School of Management, Purdue University, West Lafayette, IN 47907, USA; oonosu@purdue.edu

**Keywords:** self-identity, cultural immersion, perspective transformation

## Abstract

This study focused on understanding the cultural immersion experience of students who participated in Study Abroad Programs (SAP) and Global Service Learning Programs (GSL). The study looked at how the immersion experience impacted the participants’ view of self and others upon re-entry into their local community. Specifically, we applied the perspective transformation theoretical framework to analyze the extent to which participation in cultural immersion programs transforms students’ perceptions of self and others. The analysis of the semi-structured interviews suggested that by engaging in intentional immersion and guided reflections, participants became aware of the need to reevaluate their perspectives, expectations, and assumptions about self and others. We also found that there were differences in the way participants experienced the cultural immersion process and the impact it had on cultural awareness and self-identity.

## 1. Introduction

In gauging the effectiveness of cultural immersion program, much of the emphasis has been on measuring the development of program outcomes and competencies such as global awareness, global leadership, and cultural awareness [1,2,3,4]. Others have focused on understanding the effect of structural factors such as program design, content, and outcomes [5,6,7]. While there is a necessity to continue to measure program outcomes, researchers have also alluded to the need to broaden current research to include the impact of process factors on cultural immersion experience [6,8].

Recently, a handful of studies have shown interest in researching the relationship that exists between cultural immersion participation and the process of identity transformation [9,10,11,12,13,14]. However, despite this growing interest, there is little or no research that has specifically studied the connection between participation in Global Service Learning, Study Abroad Program, and the transformation of perceptions of self and others. Consequently, to address the gap, this research draws on the literature of the transformative learning to better understand the cultural immersion experience, the social learning that occurs in the host environment, and the impact that the process has on transforming participants’ self-identity and their perceptions of others. This research moves beyond structural factors and examines the unique impact of participation in study abroad and global service learning on the transformation of self-identity and perceptions of others.

We begin with a brief review of the study abroad and global service learning literature, and then give an overview of the transformational learning theory and the concept of self-identity. We then describe the study method and findings. We conclude with a discussion of the implication of understanding the process of identity transformation through engagement in multicultural immersion experiences within diverse local communities.

### 1.1. Study Abroad Programs (SAP)

Study Abroad Program (SAP) frequently involves a cultural immersion experience that purposely exposes students to a cultural environment that is different from their local community [15]. SAP entails either a credit or noncredit-based educational and cultural experiences that require students to live and study in a foreign country for a specified period [16]. The three main types of SAP programs referred to in the literature are topic-focused instructor-led short-term study abroad, community engagement study abroad, and immersion semester enrollment study abroad [2]. While there are some differences in program content and structure, each of these SAP programs is similar in that it uses formal academic activities and cultural experience to foster cultural exposure.

Many colleges and universities offer various forms of these SAP programs to create opportunities for students to participate in a cultural immersion experience. A recent Institute of International Education Open Doors report shows that 332,727 students from across colleges and universities within the United States studied abroad in 2016/2017. This figure represents an increase of 2.3% in the number of students who participated in SAP from the previous year. The breakdown of this report shows that 64.6% of U.S. students participated in short-term summer programs, 33.1% in mid-length programs, and 2.3% in the long-term programs [17]. Interestingly, the majority of U.S. students who study abroad are predominantly white female undergraduate students [17]. Additionally, the students show a strong preference for short-term SAP with destinations in Europe, Latin America, the Caribbean, and Asia, with only a few participants choosing to go to destinations outside of these regions [17].

### 1.2. Global Service Learning (GSL)

Global Service Learning (GSL) is a specific type of cultural immersion experience that entails participation in a local or international experiential learning activity that integrates educational learning and critical reflections with community service [6]. An essential characteristic of the GSL programs is that it is reciprocal. The GSL program tends to connect education and community service in a way that it benefits both program participants and host communities [5,6,18,19]. One of the significant structural aspects of the GSL program is the “lived experience”. Where participants immerse themselves in the host community and work together on service projects alongside members of the host community [6]. The immersion process creates opportunities for participants to engage in reflection activities that help them to connect the lived experience with the cultural, political, and social situations in the host community within a global context [6].

Typically, the design of the global service-learning program varies as either a co-curricular global service learning, short-term global service learning, course-embedded spring break global service learning, or a curricular embedded global service learning [20]. Each of these program structures includes either a short-term, medium term, or long term cultural immersion component. The short term immersion experiences are generally between one to two weeks. The medium term immersion is between six to eight weeks, and the long-term immersion is typically between 52 weeks and one academic year [21]. The immersion experience allows students to encounter a new culture. During the cultural immersion, participants mostly stay with host families, but sometimes they also live in alternative forms of housing within the community [20].

### 1.3. Perspective Transformation Theory

The Mezirow’s [22] perspective transformation theory and Kiely’s [23] transformative service learning model, provides an excellent theoretical lens to examine the transformation of self that occurs during cultural immersion. The perspective transformation theory explains that when people encounter an unfamiliar situation, it pushes them to alter their regular pattern of thinking, and this experience might lead to the development of new perspectives. Mezirow [22,24] found that people naturally function from a set of “habitual expectations” known as perspectives. Mezirow then argues that people develop their perspectives through the lifelong process of interaction and socialization. These perspectives then become the frame that shapes their beliefs, values, ideas, and viewpoints. Whenever a person encounters a disruptive event, it triggers a transformative learning process and creates a new reference point, which causes them to reflect on their beliefs, norms, values, ideas, and expectations. Through this reflection process, people can then develop new perspectives that become the pivotal point of relating to self, others, and society [22,24]. If an individual does not encounter or experience situations that challenge perspectives, their perspective will remain unchanged. Illeris [25] further explains the process of transformative learning and the development of new self-identity. He claimed that identity involves multiple personality layers, which makes it difficult for people to alter their self-identity naturally. Illeris believes that it takes a strong reason and motivation to change self-identity. Hence, he concludes that self-Identity transformation can only happen when people experience a motivation that is strong enough to trigger transformative learning [25].

Kiely’s transformative service learning model explains how transformation occurs during global service-learning experiences [23]. Kiely argues that during global service learning, transformative learning happens in five dimensions: contextual border crossing, dissonance, personalizing, processing, and connecting. The contextual border crossing describes the personal, structural, historical, and programmatic elements that can influence the learning process that promotes or deters transformative learning [22]. The dissonance illustrates the differences between previously held assumptions and the new experience. While the personalizing represents how individuals respond to and learn from conflict. Finally, processing and connecting explain how reflective learning, allows individuals to adequately understand, empathize, and transform the learning experience into actions during and after the service engagement [23]. Mezirow perspective transformation theory and Kiely’s transformative service learning model provide a unique theoretical lens to analyze the extent to which students experience the transformation of the perception of self and others during and after participation in GSL/SAP.

### 1.4. Cultural Immersion, Transformative Learning, and Self Identity

Studies show that often when students participate in immersion activities such as GSL/SAP, the cultural exposure triggers an awareness process that can lead to the reevaluation of personal views of self and others [10,11]. For example, Ellwood [11] explored the concept of continuity and identity change based on the data from four students who studied abroad at an Australian university. He found that study abroad experience could broaden perceptions, challenge norms, and transform identity socially and personally. However, the study showed that the level of change experienced by the participants was not necessarily the same across all students because students exist as individual learners.

Additionally, the result of this study suggests that identity transformation occurrence depends on the intensity and length of cultural immersion. Expanding on this notion of cultural immersion and identity, Chan and Klayklueng [10] investigated the impact of short-term study abroad immersion in Singapore on critical cultural awareness and identity. They found that the process of immersion provided learners the access to identify, analyze, interpret, and evaluate their own culture as well as the host culture in ways that led to both the reaffirmation and reconstruction of their self-identity.

Few studies have examined the impact of service learning (SL) on students’ identity and development [26,27]. Studying the effect of power and identity on African American students, Winans-Solis [27] revealed that participation in service learning was essential in defining and navigating self-identity. Mainly, this study illustrates the importance of structured reflection and service in the processes that the students employed to deconstruct held identity and then reconstruct a new self-identity.

Similarly, a few studies have examined the role of international service learning (ISL) in facilitating students’ construction, conception, and development of self [14,28,29]. For example, Yang et al. [29] define the conception of self as a process of learning to use cultural values to construct role and actions in the social group that people belong to within their society. Using this definition of the conception of self, Yang et al., explored the concept of self in how students construct their moral, cultural, and leadership roles during and after international service learning. The evidence from the study showed that vast difference exists in the students’ conception of self, in terms of their understanding of moral, cultural, and leadership roles. They observe that the discrepancy noticed in the students’ views of self was mainly due to the difference in the levels of social engagement, personal interest, and ways in which they navigated the cultural differences during the global engagement. Other studies suggest that global service learning creates a unique environment that facilitates the transformation of self-identity [14,28,29] For instance, Mather et al. [28] discover that international service learning gives participants the context to recognize past experiences that have shaped their identity, and then offers a broader experience that serves as a new foundation to reconstruct self-identity. Although various scholars support the notion that cultural exposure promotes self-development, yet there is little research that examines the process of identity transformation during and after GSL/SAP experiences. Hence, the purpose of this study is to understand the process by which cultural immersion experience (GSL/SAP) impact the transformation of self-identity and perceptions of others

## 2. Materials and Methods

The study employed a qualitative approach to collect, analyze, and interpret data. Patton [30] argues that the qualitative research method is the most appropriate tool to answer research questions that relate to social events and personal experiences. Since the aim of this study was to understand the extent to which GSL and SAP facilitate the transformation of self-identity, semi-structured interviews, and document analysis provided an adequate method to collect the data for the study [30,31].

### 2.1. Participants

Given that this is qualitative research, we applied the Malterud, Siersma, and Guassora concept of information power to determine the adequate sample size for the study [32]. The information power concept addresses five critical aspects of study design; this includes study aim, sample specificity, use of theory, dialogue, and analysis strategy [32]. According to the information power model, a qualitative study that has a narrow aim, participants that hold specific characteristics, theory, strong dialogue, and analysis strategy will require the least number of participants [32]. As this study meets all of the items addressed in the information power model, the number of participants needed was less than what is traditionally required to determine power calculation.

We used a snowball sampling technique to recruit 26 participants for this study. Noy contends that in conducting social research, snowball sampling provides a distinct category of participants that have a rich knowledge of the subject of interest [33]. The snowball sampling method gave direct access to students and faculty members who had participated in a credit-based curriculum GSL/SAP program. The study sample comprised both students and faculty members from public and private universities in the northeastern, western, and southern regions of the United States. The participants interviewed consist of past and current students from various academic majors (International Business, Management, Pre-Engineering, Spanish, Nursing, Sociology, Education, and Global Studies Program), who had participated in a short-term cultural immersion trip within the last one to seven years. Additionally, the participants in the study varied in gender, social class, race, age, and the type of programs attended.

We duly informed all the participants about the research and ensured that participants read and signed the informed consent before scheduling the interviews. Participants interviewed included two SAP faculty members (*n* = 2), one from a private university, and the other from a public university and GSL faculty members (*n* = 4) two from private universities and two from public universities. Student participants include SAP undergraduate students (*n* = 10), four from private universities, and six from public universities. GSL graduate students (*n* = 5) all from public universities. GSL undergraduate students (*n* = 5) four from private universities and one from a public university. Table 1 gives a summary of the description of the demographics of the study participants.

### 2.2. Data Collection

After obtaining the Institute Review Board (IRB) approval for the study, the data collection process began, and it lasted for over nine months. Primarily, the data collection involved document analysis and in-depth individual interviews. For those participants who resided within the study location, the interviews took place at a private and convenient site of their choice. While for participants who lived out of the study region, the interviews happened online via Zoom. The interview sessions lasted between 45–60 min. At the start of the interview sessions, we used a question guide to initiate the conversation and then probed further for additional information. Patton [30] believes the use of the question guide is a good practice and a way to enhance the flow of information that can illuminate a deeper understanding of peoples’ experience.

The secondary data collection involved the review of program information from the brochures, program proposals, and program curriculum obtained from faculty members. We analyze these documents to collect additional information about the program structure and curriculum. Patton [30] suggests that the review of program documents provide the researcher with information that can reveal events that took place before, during, and after the program. This data collection technique gave additional information that may not ordinarily be observable or obtainable from the participants [30].

## 3. Results

During the data collection phase, 45–60 min interviews were conducted face to face or by zoom with a small purposive sample of US undergraduate (*n* = 15), graduates (*n* = 5) students, and faculty (*n* = 4) who had participated in a short-term study aboard program (SAP) or short term, credit based global service learning (GSL) curriculum. we digitally recorded the interviews and transcribed verbatim into a Microsoft Word document [30]. We then uploaded the transcripts into NVivo 11(Pro), a qualitative research software tool created by QSR International (Burlington, MA, USA) for reading, re-reading, and coding. We employed a cyclical content analysis approach to analyze the data. The initial coding involved a structural method, using the research question alignment to code. We then applied an In vivo coding, using the participants’ own words to recode the data, before finally themeing the data [31]. After the first coding, we shared the first sets of categories and themes that developed from the data with some of the faculty members that we interviewed, and based on their feedback, we re-coded the themes using the In vivo coding technique. According to Patton, this process of sharing emerging themes with experts help to enhance and refine the study findings [30]. Finally, through the process of coding, recoding, and themeing, the categories and themes that represent the views and experiences shared by the study participants emerged.

The categories and themes that emerged from the data analysis include experiencing cultural immersion (perspectives, cultural immersion), recognizing assumptions (questioning assumptions, reconstructing perspectives), and understanding self and others (redefine self and others, different and unique). Experiencing cultural immersion category represents themes that explained the participants’ cultural experience and their reactions to dissonance situations. It also discusses how, through encountering these situations, participants gained new realizations about self-identity that triggered transformative learning. The themes under the category “recognizing assumptions” described how, through the challenges, participants were able to question assumptions, values and then change their perspectives. The “understanding self and other” category refer to themes that discuss the process by which participants experienced a shift in their perspectives, which eventually resulted in the development of a new understanding of self and others.

### 3.1. Perspectives

Many participants in this study observed that stepping out of their familiar culture to experience a new culture made them realize how much of their held views about self and others reflected their social and cultural upbringing. For instance, one of the participants explained that before her global service-learning trip, she considered herself as someone that was “inclusive and looked at things from a social lens”. However, through the guided reflections process during the cultural immersion, she quickly realized the influence that her family upbringing, class status, cultural and social expectations had on the perceived lens through which she viewed others. Jane recalled an incident that happened during her trip that helped her to come to this understanding. She shared about her initial reaction to her host family in Colombia, waking up early each morning to start their day. Narrating the experience in this quotation:

The thing I think got me frustrated the most, and I don’t want to say the lack of concern, but they wake up super early like 4 a.m., everybody starts early, I don’t know why. My room was near the kitchen, the computer was just right there, and my room was this little panel so I could literally hear everything, nobody was trying to be quiet early in the morning. You can hear them, banging pots and pans, talking out loud, they were not trying to whisper. There were times that I would be woken up at 4 a.m., and I was like, I cannot do this; I was so frustrated. Even with my roommate in school, if you get up before somebody else does, it is normal for you to be quiet, it is normal for you not to make noise. Here you don’t try to tiptoes when others are sleeping, that annoyed me so much.

From this experience, it became apparent that often, perceptions, assumptions, and expectations about appropriate behaviors are culturally and socially construe [18]. Similarly, Gibson [34] introduces the idea of role modelling as an important aspect of how people develop their self-identity and understanding of behavior. Typically, role model are significant figures in the ones social circle from whom they learn their perception of appropriate behavior [34] Through the process of guided reflection, Jane was able to understand the reason why she felt offended by what she perceived as the host family insensitivity to her need for morning rest. Jane was interpreting the behavior of her host family through her own experiences and cultural understanding of how people should use shared space. Unlike her culture, in the host community, the expectation this that people should wake up early and participate in the daily chores as a way of bonding and building family ties. Like Jane, many other participants shared similar stories that reinforce Mezirow’s and Gibson’s theoretical notion that peoples’ perceptions of others develop from socialization and role models that influence, assumptions, values, beliefs, and experiences [22,34].

### 3.2. Cultural Immersion

During their cultural immersion, most of the SAP and GSL participants recognized that you could never fully anticipate the challenges of experiencing a new culture. The GSL and SAP participants observed that physically being in a location is much different from reading about it in a book. A reoccurring theme from the interviews points to the need to go into the cultural experience with openness and a readiness to learn from all situations. For instance, Debra reiterates the importance of openness, saying, “honestly, I believe that if you are willing to put yourself out there and not be holding on stubbornly to your [own] view of things, you will experience and learn something new”. The participants mentioned that although the pre-departure preparation was necessary, a mindset that is flexible and ready to adapt was vital for one to be able to adjust when the unexpected occurs.

Many of the participants believed that open-mindedness was essential to achieving transformative learning during and after the cultural immersion trip. Additionally, the findings suggested that the ability to reflect on the social, economic, cultural, and traditional ways of life of the people in the host location provided the participants with learning opportunities beyond what conventional education offers. For example, Douglas shared that during a two weeks GSL trip to South America, he finally grasped the concept of social status. He explained saying, “we saw a different group of people in terms of socioeconomic status, and for the first time I understood the difference in people’s status, and that experience helped me become sensitive to these things”.

Furthermore, the findings showed that the learning process in a location might be different for participants based on their racial and ethnic identity. Some participants talked about how the host community perceived minority students differently and the effect it had on the learning process. For instance, Doris shared that, “my experience as a white woman going to Jamaica will be very different than if I was going to Mexico or if I went to Peru”. She explained her reasons for thinking this way, saying that, “there was one African American student in our group, her experience was different from ours because people didn’t immediately look at her and think she was a foreigner”. Many of the minority students interviewed confirmed these assumptions. They shared that in their host location, at first, most community members related to them based on their racial and ethnic identity rather than their American Identity. The findings also suggested that the transformative learning during cultural immersion does not necessarily depend upon the length of the stay in the host community, but on the willingness and the intensity with which the participants engage with the people. For instance, one of the GSL participant explained this concept saying that: “you know, you could go to Jamaica, and you spend your entire trip surrounded by other European or Americans, never really experiencing Jamaica, I think that can happen in GSL cultural experience if you are not immersed in the host community”. Participants also described the impact of living with a host family and the effect it had on transformative learning. For example, a participant said, “the host family is huge, and it is one of the best things about the program for students, “it gives you a way in, and once your family accepts you, the community accepts you”. Another participant believes that the host family was an essential part of the cultural immersion process because it provides situations that force people to step out of their comfort zones and venture into the unfamiliar. She explained saying:

“If we had gone back to dorms, hotel rooms, or any other kind of stuff, the experience would not have affected us as deeply as it did. At least for me, it definitely wouldn’t have, because you can shut it out, you can compartmentalize it, you can shut yourself off from it, but here, there was no getting away from it”.

For others, the fact that they were sharing living space with students from diverse backgrounds was a vast learning experience. For example, Samantha, a study abroad participant, said, “I was living with about six to seven people all from different countries, all of us going through the same thing but coming from different places, this was a learning experience for me”.

### 3.3. Questioning Assumptions

Interestingly, both the SAP and GSL participants acknowledged that their immersion experience allowed them to begin to see flaws in how they view American culture in comparison to other cultures. Participants recalled that before engaging in the cultural immersion experience, they had limited knowledge about other countries. For example, Samantha shared that before studying abroad, she thought that there was only one way of doing things. She explained, “we were very much taught that the American way is the way, I went to the Netherlands, and I saw a completely different way of life, a completely different way of social policies, and government interaction with its people”. Similarly, John shared that during his GSL trip to Guatemala, he realized how polarized the American viewpoint is, saying, “We talk about American issues as if they are worldwide issues and we will only talk about global issues only if it applies to our own issues”.

For others, the experience made them question the conventional education system and the limitation it places on the ability to reflect critically on personal perspectives. For instance, one participant described this saying, “you can now look at things from an outsider’s perspective, and you see yourself and the bubble you grew up in from a different lens”. Other participants also admitted that the cultural immersion experience made it possible to see the benefit of engaging in reflective learning and the critical knowledge that develops from participating in this process.

### 3.4. Reconstructing Perspectives

Participants also described the different encounters experienced during the immersion and the transforming effect these experiences provided. The findings showed that participants who had certain preconceived expectations about their trip were more prone to anger when they encountered unexpected situations. For instance, Katelyn, one of the participants, recollected her experience with dealing with an unpleasant situation during her trip. Katelyn described the process it took for her to move from the feeling of anger and discomfort to a point where she could bring herself to learn from the situation. Katelyn shared her experience, saying:

“I remember it was like 10 p.m., and I was standing in front of my suitcase staring at it and ready to throw everything in and take the next cab to the airport, and pay whatever it cost to go back. I was so close to doing that, and then I was like, it’s ok I need to take a step back, and I need to breathe. I closed my eyes, I took a few deep breaths, and I was like, everything is going to be okay. I need to learn to let go of these expectations, I need to let go of my anger, and I need to roll with the punches like there’s nothing that is going according to plan. I was so angry that things weren’t planned. I was just like, take everything just as it is, and I remember that night I slept pretty well because I finally loosened up on the inside. I remember that after that fourth day, the time that I had there was like one of the best times that I have ever had in my whole life. Now, don’t get me wrong, I had so much more to come, but I let go of my expectations, and I realized that with that, you enjoy things, and you look for the best as they are and that in itself, is so beautiful”.

However, the findings of this study show that not all participants who experienced disorienting situations were able to turn it around into a beneficial learning experience. Some of the participants’ interviewed reacted differently in similar circumstances. These participants indicated that because of the uneasiness they felt in these problematic situations, they became turned off, and their reaction restricted their ability to learn from the experience.

### 3.5. Redefining Identity for Self and Others

Participants repeatedly spoke about how, during, and after their cultural immersion trips, they became aware that generally, all people are in search of the same fundamental human needs, such as joy, peace, and happiness, regardless of their physical difference. Participants shared that they began to realize that the similarities that exist between people are far greater than the differences. For instance, Doris shared that: “people are different only because of culture, but we are all the same, I just wish we can as a people see this truth, and then we would be able to respect one another”. Essentially, the immersion experience allowed the participants to develop the understanding that humans have cultural differences that shape the structure of their society and ways of life, but at the core, people share many similar traits. Throughout the cultural immersion, many of the participants began to notice that despite the difference in geographical location, race, and culture, humans share common characteristics. Summarizing her cultural immersion experience, one of the GSL participants explained that:

“I have come to view not only people and cultures with very different level of understanding than what I had before, but I have also come to see myself and also my country and its place in the world differently”.

### 3.6. Different and Unique

The study finding showed that on re-entry into their community, most participants continue on the path of transformation, but with time, many of the participants gradually become sucked back into their old culture. Both the GSL and SAP participants interviewed agreed that, over time, after returning to their home base, they found it challenging to keep up with the change that they began to achieve before, during, and after their immersion trip. One of the participants claimed that it was hard to keep making changes because, on return, it becomes evident that the local “culture” is a powerful force that “is ingrained in the structures all around the environment in which one live”. Another participant explained, “after you sleep off all the jet lag, it would begin to feel like you never left, your family, friends, and before you know, you just get back to your usual routine”. The findings indicated that for each of the participants, the post-immersion experience and the transformative learning process is different and unique. To maintain the transformative momentum and to keep from reverting to old behaviors, the data showed the need for participants to continue to intentionally reflect and align their perspectives and actions to transformative outcomes. One participant explained this concept saying, “I found myself resisting at first but then falling back into it, culture has such a strong effect on you that you find yourself doing things because we are not reflective of our behaviors and actions”.

Explaining the students’ post-immersion experience, Dr. Mary, one of the faculty members interviewed, believes that the learning process that occurred during the cultural immersion experience is not unilateral; this means that the outcomes will differ for each participant. She stated that “on the personal pathway that each student has to take, they will end up in different places”. Similarly, Dr. Helen explained that transformative learning is a process that involves personal decisions. She described the cultural immersion experience as a tool that each participant will have to figure out how to use. She explained this saying:

“I’m giving them some new tools and information, but, they are autonomous human beings, and it is in their place to decide the meaning it has for them, and what they want to do with it, but the tools that I give to them are good ones”.

On the contrary, Dr. Daniel, a faculty member interviewed, believes that the regression seen in their thinking and actions after the immersion experience indicates a deeper structural problem. He noted that on return from the trip, some of the participants intentionally stop engaging in reflection that will cause them to deal with disturbing realities in their local community. Hence, with time, the fundamental change witness during the immersion experience “becomes shallow and superficial, and then, it eventually fades off”.

### 3.7. Study Limitations

Since the snowball sampling approach involves using individuals to identify participants, the likelihood of interviewing a homogenous sample that might not reflect an accurate representation of the larger population exists. To limit this effect, we triangulated the process by using multiple sources to collect and analyze the data. Another source of limitation in this study was the use of a single data collection instrument. Although qualitative research allows the researcher to serve as the data collection instrument, the researcher needs to take steps to reduce the effect of personal bias. To minimize the impact of personal preference, we created analytical memos and used member checks to verify all transcribed information. This process ensured that the data analysis procedure was reflective and that the themes that emerged reflected the participants’ perspectives of their experience [31].

Finally, the variations in the structure and design in the different types of GSL/SAP programs that the participants in this study attended created another source of limitation. Only data from students who participated in a short-term credit-based curriculum GSL and SAP programs were included in this study to reduce this effect. However, because the impact of this type of experience is progressive [25], and the data for this study was collected only ones, these results provide a limited time frame to examine the impact of these transformative experiences, which is an additional limitation of the current research.

## 4. Discussion

The purpose of this study was to examine the cultural immersion process and the impact that the experience had on transforming students’ self-identity and their perceptions of others. The qualitative analysis guided by a theory of perspective transformation and self-identity revealed a variety of short-term changes in participants’ perspectives, with suggestive correlations between depth of program impact and levels of social engagement, personal interest, and ways in which participants navigates cultural difference in multicultural situations. The findings from this study provide insight into ways in which program participants transform the excitements and challenges experienced before and during cultural immersion into learning opportunities. First, many of the categories parallel work by Kiely [23] that capture the different types of transformation immersion experiences participants reported. The findings also allude to the importance of pre-departure preparation in the transformative learning that occurs before, during, and after the cultural immersion. Importantly, participants noted that the pre-departure preparation (assigned readings, reflections activities, and research about the host community) enhanced the learning process that happened at the host location. Explicitly, participants cited the pre-departure preparation as a factor in their ability to cope in unfamiliar territories. Many of the participants shared that the knowledge gained during the pre-departure reflections provided learning mechanisms that caused them to develop a unique understanding of themselves in their host community. These findings support Giles and Eyler [35] and Slimbach [36] claim that adequate preparations before the cultural engagement results in the development of knowledge and skills that are critical in fostering transformative learning.

The findings also suggest that a combination of factors such as the intensity of immersion of participant engagement in the local environment, housing, and reflective practice that exposed participants to a different way of thinking, cumulatively contributed to the transformative outcomes observed. Some participants indicated that transformative learning would not happen without the lived experience and the opportunity it provides to interact with the host community. Many of the students who participated in the GSL program stated that the service component of the program was also another effective means of connecting with their host community. This finding supports Slimbach’s [36] argument that during cultural immersion, learning does not happen in a vacuum. He suggests that during cultural immersion, participants’ character and behavior will interact with external factors, such as program design, the environment, and the host community to either restrict or expand the transformative benefits of the cultural experience [36].

Furthermore, the students conclude that the cultural experience allowed them to realize the similarities that exist among people despite cultural differences. Hence, this kind of understanding empowered and motivated many of the participants to begin to shift their perspectives of self and others from a narrow cultural view to a more global perspective. For many of the participants, going through this experience was strange and shocking, but it forced them to begin to rethink their view of self and others. For example, participants explained that the experience made them understand that people share the same desires irrespective of geographic location, language, race, and social and economic status. The observation is consistent with Kiely’s findings in his study that looked at the international service learning experience of 22 students in Nicaragua [19]. It also collaborates the assumptions of Mezirow’s transformative theory that claims that exposing a person to a disorienting situation triggers a reflection process that leads to the reassessment of assumptions and perspectives [22].

While for some participants, the cultural immersion experience signaled the start of a transformative self-identity journey that they continue to navigate on re-entry to their society, through various platforms such as career, community activism, and civic engagement. For others, upon return to their community, they struggle to continue on the path of transformative change, and over time, they began to experience a decline in their desire and interest in seeking transformative outcomes. Explaining this regression, Illeris [25] connected the concept of transformative learning to identity and argued that because identity is a complex structure, people experience change subjectively. Illeris believe that “sometimes progressive transformation can be too demanding and challenging for learners so that the outcome instead becomes withdrawal or regression, which in itself can also be a kind of transformation [25] (p. 160). To maintain the transformation and to keep from reverting the change, the findings suggest that the SAP and GSL participants need to continue to reflect and align their actions with transformative practices.

To a large extent, the categories and themes that emerged from the data support the claim that cultural immersion engagement such as SAP/GSL creates an enabling environment that helps to shape people’s perspectives and identity [29]. Interestingly, the findings also showed that based on factors such as educational level, age, race, ethnicity, social class, and gender, people experience cultural immersion and its impact differently. These findings are consistent with Kiely’s work that shows that during GSL, personal, structural, historical, and programmatic elements can influence the process in a way that promotes or deters transformative learning [23].

## 5. Conclusions

Generally, the findings from this study validate other research that suggests that cultural immersion experience facilitates the construction, conception, and the development of self- identity [14,19,28,29,37,38]. The study further offers an in-depth insight into the process and pathways through which the transformation of perception and self-identity occurs before, during, and after cultural immersion experience. These findings are significant because it provides useful information that can help faculty members and program partners to design programs effectively. It also offers future program participants the needed tools to engage in transformative learning before, during, and after cultural immersion experience. Although the findings from this study mostly speak to the benefits of cultural immersion in transforming self-identity, it equally shows the need for more research in this area that will further develop this concept and explore the various triggers associated with meaningful transformation for self-identity together with key educational outcomes. Additionally, to expand this knowledge, future study can focus on understanding the potential psychological outcomes for the other stakeholders such as host families, host communities, program service providers, and the collaborators from the host societies who are equally involve in the transformative learning process.

## Figures and Tables

**Table 1:** Participants Demographics information using pseudonyms.

Pseudonyms	Age	Educational Level	Gender	Race	Social Class
Alex	26–49	Faculty	Male	White	Middle Class
Helen	50–64	Faculty	Female	White	Middle Class
Mary	50–64	Faculty	Female	White	Middle Class
Daniel	50–64	Faculty	Male	White	Middle Class
Rebecca	26–49	Graduate	Female	White	Middle Class
Doris	26–49	Graduate	Female	White	Middle Class
Donald	26–49	Graduate	Male	White	Middle Class
Douglas	18–25	Graduate	Male	White	Middle Class
Dennis	26–49	Graduate	Male	White	Working Class
Donna	18–25	Undergraduate	Female	Biracial	Middle Class
John	18–25	Undergraduate	Male	Asian	Middle Class
Joe	18–25	Undergraduate	Male	White	Middle Class
Jane	18–25	Undergraduate	Female	White	Middle Class
Amy	18–25	Undergraduate	Female	Hispanic	Middle Class
Jackson	50–64	Faculty	Male	Asian	Middle Class
Gabby	50–64	Faculty	Female	White	Middle Class
Dorothy	18–25	Undergraduate	Female	African American	Middle Class
Cassidy	18–25	Undergraduate	Female	Biracial	Middle Class
Brianna	18–25	Undergraduate	Female	White	Middle Class
Lilly	18–25	Undergraduate	Female	Latino	Middle Class
Collins	18–25	Undergraduate	Male	White	Middle Class
Samantha	18–25	Undergraduate	Female	White	Middle Class
Ella	18–25	Undergraduate	Female	Asian	Middle Class
Debora	18–25	Undergraduate	Female	White	Middle Class
Elijah	18–25	Undergraduate	Male	African American	Working Class
Katelyn	18–25	Undergraduate	Female	Asian	Middle Class
